# The Impact of Chronic Tobacco Smoking on Retinal and Choroidal Thickness in Greek Population

**DOI:** 10.1155/2016/2905789

**Published:** 2016-01-14

**Authors:** Marilita M. Moschos, Eirini Nitoda, Konstantinos Laios, Dimitrios S. Ladas, Irini P. Chatziralli

**Affiliations:** ^1^1st Department of Ophthalmology, Medical School, National & Kapodistrian University of Athens, 154 Mesogion Street, 11527 Athens, Greece; ^2^1st Department of Ophthalmology, Medical School, National & Kapodistrian University of Athens, 75 Micras Asias, Goudi, 11527 Athens, Greece; ^3^First Department of Ophthalmology, History of Medicine Department, Medical School, National & Kapodistrian University of Athens, 75 Micras Asias, Goudi, 11527 Athens, Greece

## Abstract

*Aim*. To investigate the effect of more than 25-year cigarette smoking on choroidal and retinal thickness, using spectral domain optical coherence tomography (SD-OCT).* Methods*. Thirty-one smokers and 25 age- and sex-matched nonsmokers, serving as control group, were submitted to slit-lamp biomicroscopy and dilated fundoscopy, SD-OCT, measurements of intraocular pressure (IOP), central corneal thickness (CCT), and axial length (AL). Heidelberg Spectralis was used to calculate choroidal thickness (CT), ganglion cell complex (GCC), outer retina layers (ORL), and macular thicknesses (MT).* Results*. The smokers' group consisted of 17 males and 14 females with mean age of 57.8 ± 4.5 years, while the controls' group consisted of 14 males and 11 females with mean age of 68.0 ± 4.1 years. CT and GCC thicknesses were significantly reduced in smokers compared to control group. The differences in thicknesses of ORL were marginally significant between two groups. The measurements of MT, IOP, CCT, and AL had the same distributions between smokers and nonsmokers.* Conclusions*. Tobacco smoking seems to result in thinner choroid and retina compared to nonsmokers. This is the first study in literature that investigates the anatomical effect of smoking for more than 25 years on the choroid and retina.

## 1. Introduction 

Tobacco smoking remains a leading cause of preventable death worldwide, being responsible for 2-fold and 1.3-fold increase of mortality in current and former smokers, respectively [1]. According to World Health Organization (WHO), tobacco kills around 6 million people per year, including more than 5 million deaths caused by direct tobacco use and approximately 600 thousand deaths of nonsmokers being exposed to second-hand smoke. The duration of smoking and the number of cigarettes are strongly related to high morbidity and mortality, being observed in both men and women, especially after 60 years [1]. The tobacco mortality is associated with lung cancer (85%), chronic obstructive lung disease (70%), and cardiovascular disease (10%) [2]. The effect of smoking on skin, soft tissues, and reproductive and immune system is described by the increased prevalence of bacterial infections, including pneumonia, tuberculosis, surgical complications, periodontitis, and sepsis as well as fungal infections, hepatitis B and hepatitis C, common cold, and HIV in smokers [3]. In addition, smokers exhibit impaired wound healing, collagen production, and bone mineral density, which is related to higher fracture risk [3].

The modification of immune responses caused by tobacco is a possible explanation for the implication of smoking in ocular inflammation, including uveitis, scleritis, ocular surface, and Graves' disease [4, 5]. Furthermore, cataract, age-related macular degeneration (ARMD), diabetic retinopathy, contact lens-related keratitis, and retinal (branch or central) vein occlusion are sight-threatening diseases, whose pathogenesis and progression are modified by cigarette consumption [5, 6]. The choroid is a 3-dimensional structure, composed of an interconnected network of blood vessels, which is essential for the normal blood supply and function of retinal pigment epithelium, outer retina, and the preliminary portion of the optic nerve. The microvascular substrate of diabetes mellitus is possibly correlated with the thinning of subfoveal and temporal choroid in patients with diabetes and microalbuminuria [7]. Bayhan et al. noted that the choroid located 1.5 mm and 3.00 mm nasal to the fovea and nasal and superior retinal nerve fibre layer (RNFL) display reduced thickness in patients with severe obstructive sleep apnoea syndrome compared to healthy individuals [8].

Subfoveal choroidal thickness (SFCT) has been found to be thinner in patients with chronic heart failure and rheumatoid arthritis and increased in hypothyroid patients [9–11]. The consumption of ethanol results in increased choroidal thickness during the first 60 min, followed by a reduction in the next 120 min [12]. Sansom et al. noted that systolic blood pressure and ocular perfusion pressure were modestly and negatively correlated with SFCT [13]. Choroidal vascular changes and retinal ischemia possibly explain the choroidal thinning of the optic disk in patients having unilateral acute primary angle closure attacks [14]. Ohsugi et al. highlighted that the thick choroid observed after cataract surgery was negatively correlated with intraocular pressure (IOP) and axial length [15]. Usher syndrome type 2 and early and exudative ARMD have been implicated in choroidal thinning, whereas polypoidal choroidal vasculopathy and central serous chorioretinopathy have been related to increased choroidal thickness [16–18]. The axial length, IOP, posterior staphyloma, and chorioretinal atrophy significantly represent addition ocular factors affecting peripapillary choroidal thickness [19]. Wei et al. noted that SFCT in people around 65 years is decreased with age (4 *μ*m per year of age) and myopia (15 *μ*m per D of myopia) [20].

The design of this study was based on the great impact that systemic and ocular diseases seem to have on choroidal vasculature, affecting therefore the outer retina and the optic nerve. The aim of this study is to investigate for the first time in literature the effect of more than 25-year cigarette smoking on choroidal and retinal thickness, using SD-OCT.

## 2. Material and Methods

### 2.1. Patients

Thirty-one smokers and 25 age- and sex-matched nonsmokers, serving as control group, were included in this cross-sectional randomized study, which was conducted at the 1st University Eye Clinic of General Hospital of Athens G. Gennimatas. All participants were submitted to slit-lamp biomicroscopy and dilated fundoscopy and optical coherence tomography (OCT) as well as measurements of intraocular pressure (IOP) (Goldmann applanation tonometry), central corneal thickness (CCT), and axial length (AL). The CCT and AL were calculated using A-scan ultrasonography (Ocuscan RxP Alcon). The study was performed in accordance with the tenets of the Declaration of Helsinki and the protocol used was approved by the ethics committee of the University Hospital. Written informed consent was obtained from all participants.

Participants with any ocular disease that prevents the examination of the cornea and retina, with a history of any ocular surgery, ocular or systemic disease, or taking any medication within the last 3 months in both groups, with a history of smoking or alcohol intake in the control group, and with a history of alcohol intake in the smokers group were excluded from the study.

Patients with 25–55 years of age, spherical refractive error smaller than 3 diopters (D), cylindrical refractive error smaller than 1 D, best-corrected visual acuity of 20/20 or better, no history of chronic ocular disease, normal blood pressure, and normal blood parameters were included (blood count, clinical chemistry, and thyroid function test). It was ascertained that the participants in the control group were not passive smokers and were not exposed to cigarette smoke at home or work. The standard pack year method was used to measure the incidence and degree of smoking and participants with minimum 25 years of smoking history and at least one pack of cigarettes per day for 1 year were included. The subjects who cannot meet any of the screening criteria were excluded.

### 2.2. OCT Measurements

Measurements of the macular parameters and choroidal thickness were performed by the same well-trained and experienced operator, using Heidelberg Spectralis (Spectralis HRA + OCT, Heidelberg Engineering, Heidelberg, Germany). This device combines a spectral domain OCT and a confocal scanning laser ophthalmoscope in a single instrument. The acquisition rate of the Spectralis OCT is 40,000 A-scans per second. Its optical depth resolution is 7 *μ*m, the digital depth resolution is 3.5 *μ*m, and the transverse and axial resolutions are 20 *μ*m and 5 *μ*m, respectively. During the procedure on the Spectralis OCT, subjects were asked to fixate on an internal fixation target to increase the chance of a well-centered scan at the fovea. The subjects were not repositioned nor was the instrument realigned during the whole scanning procedure, in order to keep the measurement conditions as constant as possible. Before examination, the pupils were dilated with drops containing 0.5% tropicamide and 2.5% phenylephrine.

The images of the choroid were acquired based on the enhanced depth imaging (EDI) OCT method, as already described by Margolis and Spaide [21]. The instrument was placed close enough to the eye to obtain inverted images, which were averaged using the automatic averaging and eye tracking features. Seven sections, each comprised of the 100 averaged scans, were obtained in a 5 × 30-degree rectangle encompassing the macula and optic nerve, and the horizontal section going directly through the center of the fovea was selected.* Choroidal thickness *(CT) was determined as the distance from the outer surface of the hyperreflective line, referred to as the RPE layer, to the hyperreflective line of the inner sclera border. The choroidal thickness in subfoveal area and at 1500 and 3000 microns from the center of the fovea in areas of superior, temporal, inferior, and nasal quadrants was recorded. The resolution of the CT image is impaired in highly myopic elongated eyes and poorly fixating patients.

The* macular thickness *(MT) in the Spectralis OCT was calculated as the distance between the first signal from the vitreoretinal interface and the signal from the outer border of the RPE. To analyze MT, a software algorithm of the Spectralis OCT interpolating thickness of the area between the scans was used. It provides a circular map analysis in which the average thickness is displayed as a colour code or numeric values in nine ETDRS (Early Treatment Diabetic Retinopathy Studies). The ETDRS map consists of three concentric rings with diameters 1, 3, and 6 mm, known from Stratus OCT.

The* ganglion cell complex *(GCC) scan protocol was used to measure the macular GCC thickness from the inner limiting membrane (ILM) to the inner plexiform layer (IPL). This protocol consists of 1 horizontal linear scan 7 mm in length and 15 vertical linear scans 7 mm in length at 0.5 mm intervals. To achieve the best coverage possible within the temporal region, the GCC protocol centered the scan at 1 mm temporal to the center of the fovea. Also, the* outer retina layers *(ORL) thickness measurements were obtained from the same scan protocol, measured from the IPL to the outer RPE. The image of the best visualization was selected for each of the eyes in smokers and control groups and the mean value of both eyes was used for each variable.

### 2.3. Statistical Analysis

The statistical program IBM SPSS Statistics 22.0 was used for the data analysis. Descriptive analysis was carried out for all parameters, including the age, the gender, the CT, GCC, ORL, MT, IOP, CCT, and AL. Nonparametric analysis Kolmogorov-Smirnov was used to check the normal distribution of the variables. The possible gender differences among groups were estimated using the chi-squared test, whereas independent samples *t*-test and Mann-Whitney test were applied to identify the possible differences in means of the quantitative variables between two groups (Mann-Whitney test was used when there was no indication of normal distribution either after Kolmogorov-Smirnov analysis or after Levene's test for equality of variances). Correlations among CT, GCC, and ORL were based on Spearman's coefficient. Furthermore, partial correlation was made among the three aforementioned parameters to exclude the possible effect of age and group.

## 3. Results 

### 3.1. Demographics

Thirty-one smokers and 25 healthy nonsmokers, serving as control group, were recruited in this study. The smokers' group consisted of 17 males and 14 females with mean age of 57.8 ± 4.5 years, ranging from 51 to 69 years. Controls' group consisted of 14 males and 11 females with mean age of 68.0 ± 4.1 years ranging from 62 to 74 years. The distribution of age is normal among participants, whereas gender's distribution is not, according to Kolmogorov-Smirnov test. There are no differences in distribution of gender between two groups (chi-squared test: *P* = 0.931) but there are differences in age (independent samples *t*-test: *P* < 0.001, Levene's test: *P* = 0.788).

### 3.2. Evaluation of Choroidal and Macular Thickness

The descriptive statistics of macular thickness (MT) and choroidal thickness (CT) as recorded in subfoveal, inner and outer nasal, temporal, superior, and inferior quadrants are presented in Table 1. The differences in means of choroidal thickness in subfoveal area and all quadrants are statistically significant among two groups, revealing thinner choroid in smokers (Mann-Whitney tests, Table 1). On the other hand, the distribution of macular thickness is the same between two groups (Mann-Whitney test, Table 1).

### 3.3. Assessment of Ganglion Cell Complex, Outer Retina Layers Thicknesses, and Ocular Features

The recorded data of ganglion cell complex (GCC) and outer retina layers (ORL) thicknesses in smokers and nonsmokers groups are displayed in Table 2, along with the differences in means of these parameters. Both superior (sGCC) and inferior GCC (inGCC) are thicker in control subjects than in smokers (independent samples *t*-test and Mann-Whitney test, resp., Table 2). Superior (spORL) and inferior (inORL) ORL data reveal that the outer retina layers are slightly thicker in smokers compared to control group, but the differences are marginally significant between two groups (Mann-Whitney test, resp., Table 2). The measurements of IOP, CCT, and AL are presented in Table 3 for both groups. The distributions for all three are the same between smokers and nonsmokers (independent samples *t*-tests for IOP and AL, Mann-Whitney test for CCT, Table 3).

### 3.4. Correlations of Ocular Data with Age, IOP, CCT, and AL

Age was positively, strongly, and significantly correlated with choroidal thickness in all quadrants, while the correlation with the subfoveal thickness was moderate but significant too (Table 4). Age was also moderately and positively correlated with inferior ganglion cell complex (inGCC), but no statistically significant correlations were observed for superior ganglion cell complex (sGCC) and superior and inferior outer retina layers (spORL and inORL, resp.) thicknesses (Table 4). Moreover, AL was weakly and positively correlated with inner (1.5 mm) superior choroidal thickness (ISCT) and superior ganglion cell complex (sGCC) thickness, applying partial correlations and counting the possible effect of age and group, but the results were not significant for the rest choroidal and retinal variables (Table 5). No statistically significant correlation was detected among MT, IOP, and CCT and choroidal and retinal thickness measurements, using again the partial correlations tests (Table 5).

## 4. Discussion 

In our study we investigated for the first time the effect of 25 years or more of cigarette smoking on choroidal and retinal thicknesses, comparing the OCT measurements of 31 smokers and 25 nonsmokers participants. We noted that the choroidal thickness in subfoveal, nasal, temporal, superior, and inferior regions was reduced in smokers compared to the control group and the differences were statistically significant among participants. Similarly, the ganglion cell complex was significantly thinner in smokers than in controls, while the differences in thicknesses of outer retina layers were marginally significant between two groups. Furthermore, age was positively, strongly, and significantly correlated with choroidal thickness in all quadrants, while the correlation with the subfoveal thickness and inferior ganglion cell complex (inGCC) was moderate but significant too. The measurements of MT, IOP, CCT, and AL had the same distributions between smokers and nonsmokers.

Our findings agree with the recent study by Sigler et al. who also supported that, besides ARMD lesions, cigarette smoking may be also related to the choroidal thinning [22]. Moreover, they correlated the decreased choroidal thickness with the duration of smoking [22]. Similarly, Sýzmaz et al. noted that choroidal thickness is reduced 1 and 3 hours following cigarette smoking compared to the one of nonsmokers [23]. On the other hand, Ulaş et al. did not reveal any significant difference in retinal thickness and choroidal thicknesses between smokers and nonsmokers [24]. However, they noted that choroid was thicker in smokers the first 5 minutes after cigarette consumption, but it returned to baseline levels after 1 hour [24]. Furthermore, Dervişoğulları et al. did not find any significant difference between smokers and control subjects neither in choroidal thickness nor in ocular pulse amplitude (OPA), which represents an index of choroidal perfusion [25]. No statistical differences in age, spherical equivalent (SE) values of refractive errors, IOP, OPA, CCT, and AL were noted between the two groups [25]. In addition, the same study group revealed that mean, superior, and inferior RNFL were thinner in smokers than control group, but ganglion cell-inner plexiform layer complex (GCIPL) exhibited no significant difference between two groups [26]. Demirci et al. supported that cigarette smoking may cause significant RNFL thinning in patients with migraine [27].

Wimpissinger et al. measured submacular choroidal and optic nerve head blood flow, fundus pulsation amplitudes, and retinal vessel diameters during and after carbogen (5% CO_2_ and 95% O_2_) inhalation by smokers and healthy volunteers [28]. Both choroidal blood flow and fundus pulsation amplitudes were increased only in nonsmokers after the carbogen breathing, whereas optic nerve head blood flow and retinal vessel diameters were reduced in both groups. No differences after carbogen inhalation were detected either for systolic, diastolic, and mean arterial blood pressure or for pulse rate and blood gas values between two groups. The different response of choroidal and optic nerve head blood flow was attributed to the fact that the former is sensitive to Pco2 changes while the latter is dependent on oxygen changes. Moreover, the abnormal response of choroidal vascularity was associated with impaired endothelial function induced by the free radical burden of cigarette smoke and a dysfunctional nitric oxide synthase III (NOS III) in chronic smokers [28]. Furthermore, endothelial dysfunction and the following arterial stiffness have been related to the synergistic effect of smoking with inflammatory molecules of interleukin-6 (IL-6), C-reactive protein (CRP), and cyclooxygenase-1 and cyclooxygenase-2 (COX-1 and COX-2) [29, 30].

Nicotine seems to increase the extent and severity of choroidal neovascularization (CNV), possibly favoring PDGF-mediated proliferation of choroidal smooth muscle cells [31]. Garhöfer et al. noted that flicker induced vasodilation and raise in blood flow of retinal veins were eliminated in chronic smokers compared to nonsmokers [32]. However, no significant differences were identified in flicker induced hemodynamic responses of retinal arteries between both groups. These results were also explained by smoking-induced endothelial dysfunction [32]. Withal, Rose et al. observed that young, healthy smokers exhibit reduced retinal vascular reactivity to normoxic hypercapnia compared with healthy controls [33]. Cigarette smoking and increased age have been associated with low blood velocity of ophthalmic artery, while systolic blood pressure has been positively correlated with velocities in the ophthalmic artery and the central retinal artery [34]. These effects are the result of interaction of nicotine-induced peripheral vasoconstriction and vasodilation caused by carbon monoxide in smokers [34].

In our study we concluded that choroidal and ganglion cell complex thicknesses were significantly reduced in chronic smokers for more than 25 years compared to control subjects, while the differences in outer retina layers were marginally significant between two groups. The measurements of MT, IOP, CCT, and AL had the same distributions between smokers and nonsmokers. These changes are possibly attributed to the endothelial dysfunction and the impaired choroidal and retinal vascular reactivity induced by cigarette smoking.

## Figures and Tables

**Table 1:** Choroidal thickness (CT) and macular thickness (MT) data in both groups.

Variables	Controls	Smokers	*P* value
	Mean (SD) median	Mean (SD) median	
Subfoveal CT (SCT)	257.0 (23.4) 256.0	229.1 (10.2) 231.5	<0.001
CT 1.5 mm nasal (INCT)	226.2 (19.9) 223.5	182.3 (8.2) 182.5	<0.001
CT 1.5 mm temporal (ITCT)	236.3 (18.8) 236.5	192.6 (5.8) 193.5	<0.001
CT 1.5 mm superior (ISCT)	241.1 (21.6) 238.0	211.4 (9.2) 211.0	<0.001
CT 1.5 mm inferior (IICT)	238.7 (22.7) 242.0	197.4 (9.4) 196.5	<0.001
CT 3.0 mm nasal (ONCT)	195.0 (21.3) 193.0	153.6 (11.0) 156.5	<0.001
CT 3.0 mm temporal (OTCT)	216.3 (17.5) 212.0	180.1 (6.9) 179.5	<0.001
CT 3.0 superior (OSCT)	230.8 (16.2) 228.0	203.4 (9.0) 202.5	<0.001
CT 3.0 inferior (OICT)	207.8 (18.6) 211.5	177.9 (12.5) 175.0	<0.001
Macular thickness (MT)	160.2 (7.1) 160.0	161.1 (9.4) 163.0	0.541

**Table 2:** Measurements of ganglion cell complex (GCC) and outer retina layers (ORL) thicknesses in smokers and nonsmokers groups.

Variables	Controls	Smokers	*P* value
	Mean (SD) median	Mean (SD) median	
GCC superior (sGCC)	95.4 (5.1) 95.0	91.5 (3.9) 91.5	0.002
GCC inferior (inGCC)	103.7 (7.0) 103.5	97.0 (6.9) 97.0	<0.001
ORL superior (spORL)	168.1 (4.5) 167.8	170.9 (2.6) 171.5	0.048
ORL inferior (inORL)	162.9 (4.2) 161.5	164.1 (2.7) 164.0	0.036

**Table 3:** Data of ocular features: intraocular pressure (IOP), central corneal thickness (CCT), and axial length (AL).

Variables	Controls	Smokers	*P* value
	Mean (SD) median	Mean (SD) median	
IOP (mmHg)	15.6 (1.5) 15.5	15.8 (1.8) 15.5	0.733
CCT (*μ*m)	547.4 (16.1) 546.0	547.6 (15.2) 548.0	0.921
AL (mm)	23.5 (0.6) 23.6	23.5 (0.6) 23.5	0.981

**Table 4:** Correlations of age with choroidal and retinal thicknesses parameters.

AGE	SCT	INCT	ONCT	ITCT	OTCT	ISCT	OSCT	IICT	OICT	sGCC	inGCC	spORL	inORL
Spearman's coefficient	0.461	0.672	0.6	0.774	0.733	0.617	0.604	0.704	0.607	0.152	0.356	−0.141	−0.142
*P* value	<0.001	<0.001	<0.001	<0.001	<0.001	<0.001	<0.001	<0.001	<0.001	0.262	0.007	0.301	0.295

**Table 5:** Correlations of MT, IOP, CCT, and AL with choroidal and retinal thicknesses parameters.

Control variables: group, age		MT	IOP	CCT	AL
SCT	Correlation's coefficient	0.033	0.238	0.170	0.319
	*P* value	0.811	0.084	0.218	0.019
INCT	Correlation's coefficient	0.200	0.139	0.036	0.152
	*P* value	0.147	0.316	0.796	0.274
ONCT	Correlation's coefficient	0.049	0.258	−0.008	0.060
	*P* value	0.726	0.060	0.954	0.667
ITCT	Correlation's coefficient	0.116	0.258	0.032	0.150
	*P* value	0.404	0.059	0.816	0.278
OTCT	Correlation's coefficient	0.145	0.157	−0.108	0.158
	*P* value	0.295	0.258	0.438	0.253
ISCT	Correlation's coefficient	0.161	0.261	0.026	0.273
	*P* value	0.245	0.057	0.853	0.046
OSCT	Correlation's coefficient	−0.001	0.206	0.077	0.184
	*P* value	0.994	0.135	0.582	0.183
IICT	Correlation's coefficient	0.140	0.197	−0.043	0.252
	*P* value	0.314	0.153	0.757	0.066
OICT	Correlation's coefficient	0.071	0.104	−0.152	0.153
	*P* value	0.608	0.454	0.271	0.268
sGCC	Correlation's coefficient	0.018	−0.022	0.234	−0.318
	*P* value	0.899	0.875	0.088	0.019
inGCC	Correlation's coefficient	−0.112	0.050	0.115	−0.071
	*P* value	0.420	0.721	0.408	0.612
spORL	Correlation's coefficient	0.032	0.161	0.030	0.068
	*P* value	0.817	0.244	0.828	0.624
inORL	Correlation's coefficient	0.379	0.174	0.010	−0.034
	*P* value	0.005	0.207	0.944	0.804
